# Acknowledgment to Reviewers of *Sports* in 2020

**DOI:** 10.3390/sports9020020

**Published:** 2021-01-24

**Authors:** Sports Editorial Office

**Affiliations:** MDPI AG, St. Alban-Anlage 66, 4052 Basel, Switzerland

Peer review is the driving force of journal development, and reviewers are gatekeepers who ensure that *Sports* maintains its standards for the high quality of its published papers. Thanks to the cooperation of our reviewers, in 2020, the median time to first decision was 18.7 days and the median time to publication was 2.6 days. The editors would like to express their sincere gratitude to the following reviewers for their precious time and dedication, regardless of whether the papers were finally published:
Abad Robles, Manuel TomásLiu, ChiangAbalasei, Aurelia BeatriceLo Monte, Attilio IgnazioAdsuar Sala, José CarmeloLockwood, ChrisAfonso Neves, JoséLogan-Sprenger, Heather M.Aguilar, Miguel PicLombardo, MauroAmăricăi, Elena ConstantaLópez Sánchez, Guillermo FelipeAmbrozy, TadeuszLópez-Fernández, JorgeAmmar, AchrafLopez-Samanes, AlvaroAnderson, MichaelLópez-Valenciano, AlejandroApostolopoulos, NikosLorenzetti, SilvioAragón Vela, JerónimoLorenzo Calvo, JorgeArdigò, Luca PaoloLukaski, HenryArnaud, GouelleLuszczyk, MarcinArsoniadis, Gavriil G.Lystad, Reidar P.Asian Clemente, Jose AntonioMadinabeitia, IkerAyuso, Juan MielgoMamen, AsgeirAziz, Abdul RashidMargaritelis, NikosBabault, NicolasMarinho, Daniel AlmeidaBadau, AdelaMarini, ElisabettaBallmann, ChristopherMarino, JosephBartolomei, SandroMarocolo, MoacirBattaglia, GiuseppeMartin, Jeffrey S.Beckham, GeorgeMartínez-Guardado, IsmaelBelcher, John R.Martinez-Navarro, IgnacioBenitez Porres, JavierMartínez-Rodríguez, AlejandroBenson, Sara ScharounMascherini, GabrieleBerecki-Gisolf, JannekeMaselli, FilippoBerger, NicolasMason, LauraBogdanis, GregoryMassaro, MarikaBonay, MarcelMate-Munoz, Jose L.Bonilla, Diego A.Mecías-Calvo, MarcosBotonis, PetrosMeerhoff, L.A.Bourgeois, Frank A.Mercer, John A.Braakhuis, AndreaMermier, Christine MBragada, Jose A.Mertens, EvelienBragonzoni, LauraMethenitis, SpyridonBrazo-Sayavera, JavierMey, JacobBricout, Véronique AurelieMichnik, Robert A.Brown, LaVerne L.Mielgo-Ayuso, JuanBrumitt, JasonMikaia, AnzorBuckner, SamualMinervini, GiuseppeBunc, VaclavMinuzzi, LucieleCabri, JanMiskulin, IvanCallaghan, SamuelMoir, Gavin LCamacho-Cardeñosa, AlbaMoon, Richard E.Camic, Clayton L.Morales-Suarez-Varela, María M.Campagna, SaraMoral-García, José EnriqueCaruso, JohnMoreno, DaniCastillo, DanielMorouço, PedroCastuera, Ruth JiménezMoya, ManuelCè, EmilianoMozos, IoanaChan, Kuei-HuiMündel, TobyChang, Chen-KangMuñoz Marín, DiegoChang, Hsiao-YunMuñoz, AnaChang, Ke-VinMurray, Steven RossChang, Shin-TsuMuyor, José MChapman, DaleNagao, HideyukiChen, NingNavarrete-Munoz, Eva MariaChen, Yung-ShengNavarro-Flores, EmmanuelChiementin, XavierNegrea, AdinaChmura, PawełNeiva, Henrique P.Cicchella, AntonioNepocatych, SvetlanaCirer-Sastre, RafelNg, KwokClemente-Suárez, Vicente JavierNicolò, AndreaClemente-Suarez, Vincente JavierNiedermeier, MartinCocca, ArmandoNiederseer, DavidCoco, MarinellaNikolaidis, Pantelis T.Collins, ChristopherNitzsche, NicoCondello, GiancarloO’Grady, MathewCook, MatthewOh, RobertCope, MichaelOlaru, Octavian TudorelCornish, StephenOliver, JonCortis, CristinaOlson, Michael W.Cramer, Joel T.Omar, Syed HarisCrocker, George H.Ortega, Félix ZuritaCuadrado-Peñafiel, VíctorØrtenblad, NielsDabbs, NicoleOxford, SamuelDadelo, StanislavasPadulo, JohnnyDankel, ScottPanek-Shirley, Leah MDe La Vega Marcos, RicardoPanidi, IoliDe Paz Fernández, Jose AntonioPanoutsakopoulos, VassiliosDe Ruiter, Cornelis JohannesParadowski, Przemysław TomaszDel Coso, JuanPardos Mainer, ElenaDencker, MagnusParnell, JillDeschenes, MichaelParraça, José Alberto Frade MartinsDi Baldassarre, AngelaPastor Vicedo, Juan CarlosDi Gangi, StefaniaPedrini, LorenzoDobrowolski, HubertPenichet-Tomas, AlfonsoDomínguez, RaulPereira, AnaDonti, AnastasiaPereira, Ana De Fátima Da CostaDonti, OlyviaPérez-Fuentes, María Del CarmenDos’Santos, ThomasPerez-Tejero, JavierDrabińska, NataliaPethick, JamieDuncan, MichaelPetisco-Rodríguez, CristinaDuranti, GuglielmoPetriczko, ElżbietaEdmonds, RohanPeyer, KarissaEstevan, IsaacPiacentini, Maria FrancescaEvans, RonaldPiekoszewski, WojciechFaghy, MarkPietraszewski, PrzemysławFelipe, José LuisPill, ShaneFeria Madueño, AdriánPitchford, NathanFernandes, John F. T.Pluta, BeataFernandes, Ricardo J.Poczta, JoannaFernández-Lázaro, DiegoPoston, BrachFerreira, Antonio PauloPrieto-Lloret, JesusFischetti, FrancescoPryor, J. LukeFominiene, Vilija BitePueo, BasilioFulford, JonathanQuinlan, JonathanFulle, StefaniaRamos, DomingoGabryś, TomaszRaquel, Aparicio-UgarrizaGaffney, ChristopherRathmacher, J.A.García Pallarés, JesúsRauter, SamoGarcía, Raquel SebioRaya-González, JavierGarcía-Soidán, Jose L.Raz, VeredGarcía-Unanue, JorgeRibeiro, Ana IsabelGareth, NyeRiemann, BryanGarnier, Yoann M.Rinninella, EmanueleGarrido, Nuno DomingosRobinson, EdwardGąsior, Jakub S.Rocha-Rodrigues, SílviaGendron, PhilippeRodriguez-Sanchez, NidiaGerasimova-Meigal, Liudmila I.Ruby, BrentGerhart, HaydenRussell, MarkGersak, GregorRutberg, StinaGibson, OliverRutkauskaite, RenataGil-Calvo, MarinaRyan, Michael J.Ginevičienė, ValentinaSagayama, HiroyukiGoins, JustinSan Juan, Alejandro F.Gomes, Thayse NatachaSantana, JefersonGómez-Baya, DiegoSantiago-Rodriguez, TashaGómez-Ruano, Miguel AngelSawyer, Brandon J.González-Badillo, Juan JoséScheid, Jennifer L.González-Sálamo, JavierSchumm, Walter R.Górnicka, MagdalenaScurati, RaffaeleGoto, KatsumasaSeiler, StephenGoulet, EricSell, Katie M.Greever, Cory J.Shaw, DaveGrosicki, GregSiedlik, Jacob A.Gross, MicahSimenko, JozefGruber, MarkusSimpson, NorahGupta, CharlotteSlimani, MaamerHa, Min-SeongSmilios, IliasHackett, DanielSo, Wi-YoungHadžić, VedranSoares, Jorge PintoHandcock, Phil J.Sole, ChristopherHanson, NicholasSon, JongsangHarandi, Vahid M.Sousa, AntónioHarnish, ChristopherSousa, Caio VictorHarrison, Andrew J.Spaniol, FrankHartanto, AndreeSponsiello, NicolaHaslacher, HelmuthStanek, AgataHassan, IbrahimStastny, PetrHatchett, AndrewStebbings, GeorginaHautala, Arto JStefani, LauraHayes, MarkŠtěpánek, LadislavHeishman, AaronStone, MichaelHernández-García, RaquelŠtrumbelj, BoroHerrmann, ChristianStruzik, ArturHew-Butler, TamaraSuchomel, TimHoffman, MartySuchomel, Timothy J.Hollerbach, BrittanySugimoto, DaiHolmstrup, MichaelSuh, HyunGyuHornsby, William GuySun, Feng-HuaHottenrott, KunoSuzuki, KatsuhikoHurst, RogerSyed-Abdul, Majid MufaqamIannetta, DaniloTaber, Christopher B.Iglesias-Soler, EliseoTaiar, RedhaIrurtia, AlfredoTakahashi, YumikoIssurin, Vladimir B.Tanaka, Tim HideakiIves, StephenTauler Riera, Pedro JoséIzawa, TetsuyaTaverna, LiviaJames, LachlanTeramoto, MasaruJaneczko, EmiliaThomas, EwanJanikowska, GrażynaTiidus, PeterJansen, Lisa T.Till, KevinJee, Yong-SeokTobina, TakuroJerome, KoralTopa, GabrielaJin, LiTorres-Sánchez, IreneJones, MargaretToubekis, ArgyrisJones, PaulTravassos, BrunoJozwiak, MathieuTrecroci, AthosJunger, JanTsoukos, AthanasiosJurkojć, JacekTucker, Wesley J.Kagawa, MasaharuUeberschär, OlafKarlsteen, MagnusUsuelli, FedericoKaviani, MojtabaValdivia-Moral, PedroKellis, EleftheriosValenzuela, Pedro LKemper, HanVan De Tillar, RolandKephart, WesleyVan Der Zwaard, StephanKerhervé, Hugo A.Van Kann, DaveKezele, Tanja GrubićVann, ChristopherKibler, Ben W.Varillas, DavidKim, Hyun-DuckVeilleux, Louis-NicolasKim, JisuVescovi, Jason D.Kim, KijinVicente João, PauloKingsley, J. DerekVieira, Taian M.Kipp, KristofWagle, JohnKlatte-Schulz, FrankaWaller, MichaelKlein, MarkusWaśkiewicz, ZbigniewKlekiel, TomaszWełnicki, MarcinKlobučar, HrvojeWerner, IngeKnechtle, BeatWest, DanielKonarski, JanWillems, Mark Elisabeth TheodorusKoryak, Yuri A.Williford, HenryKostrzewa-Nowak, DWinchester, LeeKrzysztofik, MichałWirnitzer, KatharinaKuan, GarryWitman, MelissaKump, DavidWon, DoyeonKusy, KrzysztofWu, Pei-FungKwak, Yi-SubXia, GuobinLa Torre, AntonioYanik, Elizabeth L.Landram, Michael JYou, Ren-InLane, Michael TimothyZabaloy, SantiagoLaurin, JérômeZajc, MateaLavender, AndrewŻebrowska, AleksandraLee, HaneulZelei, AmbrusLee, Jung-HoonZemková, ErikaLevitan, JacobZhou, ShiLin, Meng-TingZiemann, EwaLindsay, AngusZignoli, AndreaLinthorne, Nicholas P.Zubac, Damir

